# Transcriptomic Insights Reveal PRTFDC1 as a Novel Regulator of Myogenic Differentiation in Sujiang Pig Satellite Cells

**DOI:** 10.3390/vetsci12121197

**Published:** 2025-12-14

**Authors:** Li Zhang, Xiaowei Ye, Suyi Sun, Lei Zhang, Yixin Gu, Shinuo Cao, Mo Zhou, Weixiang Sun, Changyao Fu, Qingqing Zhang, Mei Li, Ziyue Xu, Wei Miao, Qinse Xu, Shanyuan Zhu

**Affiliations:** 1 Engineering Technology Research Center for Modern Animal Science and Novel Veterinary Pharmaceutic Development, Jiangsu Key Laboratory for High-Tech Research and Development of Veterinary Biopharmaceuticals, Jiangsu Agri-Animal Husbandry Vocational College, Taizhou 225300, China; lzhang@jsahvc.edu.cn (L.Z.); shinuo_cao@jsahvc.edu.cn (S.C.); mo_zhou@jsahvc.edu.cn (M.Z.); sunwx@jsahvc.edu.cn (W.S.); 2 School of Biopharmaceuticals, Jiangsu Agri-Animal Husbandry Vocational College, Taizhou 225300, China; 202260017@jsahvc.edu.cn (X.Y.); 2300790314@jsahvc.edu.cn (S.S.); 2400790306@jsahvc.edu.cn (Y.G.); 2300790304@jsahvc.edu.cn (C.F.); 2300790318@jsahvc.edu.cn (Q.Z.); 2300790311@jsahvc.edu.cn (M.L.); 2021010402@jsahvc.edu.cn (W.M.); 3 School of Modern Animal Science, Jiangsu Agri-Animal Husbandry Vocational College, Taizhou 225300, China; leizhang@jsahvc.edu.cn; 4 Jiangsu Jiangquhai Pig Breeding Co., Ltd., Taizhou 225300, China

**Keywords:** Sujiang pig, skeletal muscle satellite cells (MuSCs), mRNA sequencing (mRNA-Seq), differentiation, PRTFDC1, cGAS-STING signaling pathway

## Abstract

Sujiang pigs, a high-quality local Chinese breed, represent a valuable model for investigating muscle development and improving meat production through genetic selection. Skeletal muscle satellite cells (MuSCs) are essential regulators of muscle growth, with differentiation tightly controlled by specific genes and signaling pathways. In this study, MuSCs were isolated from the gastrocnemius muscle and subjected to mRNA sequencing during proliferation (GM) and differentiation stages (DM1, DM2, and DM4, collectively referred to as DM). A total of 2790 differentially expressed genes (DEGs) were identified, including 1551 upregulated and 1239 downregulated genes. Time-series analysis revealed 16 significant expression patterns. These DEGs were primarily associated with muscle development and differentiation and were enriched in Wnt, PI3K–Akt, JAK–STAT, p53, Hippo, and Apelin signaling pathways. Among them, phosphoribosyl transferase domain containing 1 (PRTFDC1) exhibited pronounced downregulation during differentiation. Functional validation demonstrated that PRTFDC1 overexpression promoted myotube formation and upregulated *MYOD1*, *MYOG*, and *MYH1* expression, whereas knockdown significantly inhibited differentiation. Furthermore, PRTFDC1 enhanced phosphorylation of key proteins in the cGAS–STING signaling pathway. Collectively, this study elucidates the temporal transcriptional regulation of MuSC differentiation in Sujiang pigs and identifies PRTFDC1 as a novel regulatory factor, providing a molecular foundation for breeding strategies and meat quality improvement.

## Simple Summary

Sujiang pigs, an indigenous Chinese breed renowned for their meat quality, represent a valuable model for investigating muscle development and enhancing meat production through genetic selection. In this study, we integrated phenotypic analysis with transcriptome sequencing to systematically characterize dynamic gene expression patterns and regulatory networks in Sujiang pig skeletal muscle satellite cells (MuSCs) during proliferation (GM) and successive differentiation stages (DM1, DM2, DM4, collectively referred to as DM). For the first time, we demonstrate that phosphoribosyl transferase domain containing 1 (PRTFDC1) positively regulates MuSC differentiation, with overexpression promoting myotube formation and upregulating myogenic marker genes, whereas knockdown impedes differentiation. Moreover, PRTFDC1 may function through the cGAS–STING signaling pathway. Collectively, these findings provide novel insights into the molecular mechanisms governing muscle growth and development in Sujiang pigs.

## 1. Introduction

Pork is a major source of meat and high-quality protein for humans. The efficiency of meat production in pigs is closely linked to the growth and development of skeletal muscle, which depends on the proliferation, differentiation, and fusion of skeletal muscle satellite cells (MuSCs) [1]. These cells, which originate from myogenic progenitor cells in the myotome, remain undifferentiated in muscle tissue after birth. They are located between the sarcolemma and basal lamina and possess significant differentiation potential [1,2,3]. In pigs, muscle fiber formation is completed by around 70 days of embryonic development, and the number of muscle fibers remains relatively constant postnatally. During the juvenile stage, skeletal muscle growth relies primarily on the continuous proliferation, differentiation, and fusion of MuSCs. In adulthood, under normal conditions, skeletal muscle mass and morphology are maintained. However, in response to muscle injury, MuSCs act as the primary stem cells, becoming activated to repair the damage [4,5,6]. Upon activation, MuSCs (as described in mouse) differentiate in a sequential manner into myoblasts (*Pax7*^+^*Myf5*^+^*Myod1*^+^) and myocytes (*Pax7^−^Myf5^−^Myod1*^+^*Myog*^+^), which then fuse to form myotubes. These myotubes further fuse and mature into new muscle fibers, a process that is regulated by a complex and tightly controlled molecular network [7,8].

Recent studies have identified an increasing number of genes and signaling pathways involved in myogenesis and MuSC differentiation [9,10,11]. However, many genes potentially implicated in these processes remain unidentified or insufficiently explored, and the precise regulatory network governing gene expression across different differentiation stages remains unclear. Therefore, comprehensive gene expression profiling of muscle differentiation across multiple species and stages is essential. To date, transcriptomic analyses of MuSC proliferation and differentiation have been performed in several species, including mice [12], geese [13], rabbits [14], goats [15,16], sheep [17], and buffalo [18], providing valuable molecular insights into muscle development. Although similar studies have been carried out in pigs, most of them have been limited to a few sampling time points, primarily focusing on the proliferative and late differentiation stages, which prevents comprehensive characterization of the dynamic transcriptional changes during satellite cell differentiation [19]. Furthermore, transcriptomic investigations of satellite cells from Chinese indigenous pig breeds remain scarce, hindering a deeper understanding of their unique regulatory mechanisms underlying muscle development [20,21].

Sujiang pigs, a breed developed through generational selection from Jiangquhai and Fengjing pigs (both Taihu pig breeds) as maternal lines and Duroc pigs as the paternal line, were officially recognized by the National Commission of Livestock and Poultry Genetic Resources of China in 2013 [22,23]. This breed is renowned for its high adaptability, tolerance to rough feed, excellent meat quality, and large litter size, making it highly valuable for both meat production and muscle growth research. However, studies on the genes associated with muscle growth and development in Sujiang pigs are limited, and the regulatory mechanisms underlying their muscle development require further investigation.

To systematically characterize the dynamic transcriptomic changes in Sujiang pig skeletal muscle satellite cells (MuSCs) during proliferation and differentiation, and to identify functional genes closely associated with muscle growth, we performed mRNA sequencing on MuSC samples from the proliferation phase and differentiation stages (DM1, DM2, and DM4, collectively referred to as DM). Analysis of differentially expressed genes (DEGs) revealed that phosphoribosyl transferase domain containing 1 (PRTFDC1) was significantly altered before and after differentiation, suggesting a potential role in muscle development. Nevertheless, the involvement of PRTFDC1 in regulating skeletal muscle growth, particularly its function during MuSC proliferation and differentiation, remains unclear. In pigs and other major livestock species, the expression patterns and functional mechanisms of PRTFDC1 are largely unexplored. Therefore, PRTFDC1 was selected as a candidate gene for functional validation to investigate its role in satellite cell differentiation and the underlying molecular mechanisms. Collectively, this study provides new insights into the molecular regulatory networks governing pig skeletal muscle development and establishes a scientific foundation for improving Sujiang pig germplasm, identifying candidate genes associated with muscle growth, and facilitating marker-assisted breeding.

## 2. Materials and Methods

### 2.1. Derivation, Cultivation, and Induction of MuSCs from Sujiang Pigs

Pig MuSCs were isolated and cultured from the gastrocnemius muscles of 11-day-old female Sujiang pigs (*n* = 3) following previously published protocols [11]. Cells were expanded in complete growth medium and seeded on Matrigel (Corning, New York, USA)-coated flasks. Fibroblasts were removed during the first passage by differential adhesion. When MuSCs reached over 90% confluence, differentiation was induced using differentiation medium, and the process was monitored for 1–5 days.

### 2.2. RNA Isolation, cDNA Library Preparation, and Sequencing

Cell samples from the induced differentiation process were collected from six-well plates, with 1 mL of Trizol reagent (Beyotime Biotechnology, Shanghai, China) added to each well for complete cell lysis. All cell samples were derived from the three 11-day-old female Sujiang pigs as described above. The experiment included four groups, GM (proliferation phase of pig MuSCs), DM1 (myocytes at day 1 of differentiation), DM2 (myocytes at day 2 of differentiation), and DM4 (myocytes at day 4 of differentiation), with three replicates per group, resulting in a total of 12 samples. These four time points were selected to capture the key stages of MuSC differentiation, including proliferation, initial myotube formation, rapid fusion, and mature myotube stages. Total RNA was extracted using the Trizol method, and RNA from other cell and tissue samples in this study was extracted following the same procedure. A total of 1 μg of RNA was used for subsequent library preparation. Poly (A) mRNA was isolated using Oligo (dT) beads, and RNA fragmentation was achieved using divalent cations and high temperature. First-strand cDNA was synthesized with random primers, followed by second-strand synthesis. The purified double-stranded cDNA underwent end repair and dA-tailing, and adapters were ligated to both ends via T-A ligation. Adapter-ligated DNA was size-selected with DNA clean beads. Each library was amplified using P5 (5′-AGATCGGAAGAGCACACGTCTGAACTCCAGTCAC-3′) and P7 (5′-AGATCGGAAGAGCGTCGTGTAGGGAAAGAGTGT-3′) primers, and PCR products were validated. Indexed libraries were then pooled and sequenced on an Illumina NovaSeq 6000 platform in 2 × 150 paired-end mode. The raw image data from sequencing were processed using Bcl2fastq (v2.20.0.422) for base calling and initial quality assessment, generating filtered raw sequencing data (Pass Filter Data) that were stored in FASTQ file format.

### 2.3. Differentially Expressed Gene (DEGs) Analysis

Differential expression analysis was performed using the DESeq2 package (v1.26.0) from Bioconductor (v3.10), running within R 3.6.3. DESeq2 is based on a negative binomial distribution model. Dispersion estimates and log2 fold changes were derived using data-driven prior distributions. DEGs were identified as those with a fold change ≥ 2 and an adjusted *p*-value (Padj) ≤ 0.05.

### 2.4. Expression Trend Analysis

To analyze DEG expression patterns, the expression values for each sample were normalized as log2 ratios: log2(v1/v0), log2(v2/v0), and log2(v3/v0), where v0 corresponds to GM as the baseline, and v1, v2, and v3 correspond to DM1, DM2, and DM4, respectively. Clustering analysis was conducted utilizing the Short Time-series Expression Miner (STEM, v1.3.11) software. Clustering results with a *p*-value ≤ 0.05 were considered statistically significant.

### 2.5. Gene Ontology (GO) and Kyoto Encyclopedia of Genes and Genomes (KEGG) Pathway Analysis

GO analysis was performed using GOSeq (v1.34.1), which annotates enriched genes with GO terms. A significant adjusted *p*-value (Padj) ≤ 0.05 was used as the threshold for identifying enriched terms. Additionally, topGO was used to generate directed acyclic graphs (DAGs) for visualization. KEGG pathway analysis was carried out to enrich significantly DEGs in relevant biological pathways. KEGG provides a collection of databases dealing with genomes, biological pathways, diseases, drugs, and chemical substances (https://www.kegg.jp/; accessed on 15 April 2024). Custom scripts were used to perform the pathway enrichment analysis.

### 2.6. Quantitative Real-Time PCR (qRT-PCR) Validation

cDNA was synthesized from RNA samples following the manufacturer’s instructions of the EasyScript One-Step gDNA Removal and cDNA Synthesis SuperMix (TransGen Biotech, Beijing, China). Quantitative PCR (qPCR) was performed using PerfectStart Green qPCR SuperMix (+Universal Passive Reference Dye) (TransGen Biotech, Beijing, China), using 18S rRNA as the internal control. Relative expression levels were calculated using the 2^−ΔΔCt^ method. Primers for all target genes, including *MYOG*, *MYH1*, *CSRP3*, *CXCL10*, *ACTC1*, *RBM24*, *SERPINE1*, *PRTFDC1*, *ESM1*, *NDRG1*, *NT5E*, *AREG*, *MYOD1*, and *PAX7*, are listed in Table S1.

### 2.7. Immunofluorescence Analysis

The cultured cell samples were washed twice with PBS and fixed with 4% paraformaldehyde for 20 min. After fixation, the cells were washed three times with PBS. Immunofluorescence blocking buffer was added to the wells and incubated at room temperature for 1 h. The blocking buffer was then aspirated, and primary antibodies were added for overnight incubation at 4 °C. After incubation, the cells were washed three times with PBS. Secondary antibodies were then added and incubated at room temperature for 1 h in the dark. The cells were washed three times with PBS in the dark. Anti-fade mounting medium containing DAPI was added to the wells to cover the cells, and samples were stored at 4 °C in the dark until microscopic observation. The following primary antibodies were used: mouse anti-PAX7 (1:100, Developmental Studies Hybridoma Bank (DSHB), Iowa City, IA, USA), mouse anti-MYOD (1:100, DSHB), mouse anti-MYOG (1:100, DSHB), mouse anti-myosin heavy chain (MYHC, 1:100, DSHB), and mouse anti-HIS tag (1:100, Solarbio, Beijing, China). The following secondary antibodies were used: goat anti-mouse IgG (H + L) cross-adsorbed secondary antibody conjugated with Alexa Fluor 488 or Alexa Fluor 555 (1:400, Thermo Fisher Scientific, Waltham, MA, USA).

### 2.8. Western Blotting

Cultured cells were collected, and total protein was extracted using RIPA lysis buffer (Beyotime Biotechnology, Shanghai, China) supplemented with 1% PMSF (Beyotime Biotechnology, Shanghai, China). Proteins were separated by SDS–PAGE on 4–20% precast gels (Yeasen Biotechnology, Shanghai, China). The following primary antibodies were used: mouse anti-PAX7 (1:1000, DSHB), mouse anti-MYOD (1:1000, DSHB), mouse anti-MYOG (1:1000, DSHB), mouse anti-MYHC (1:1000, DSHB), mouse anti-HIS tag (1:1000, Solarbio, Beijing, China), rabbit anti-PRTFDC1 (1:1000, Bioragon, Suzhou, Jiangsu, China), rabbit anti-β-TUBULIN (1:5000, Abcam, Cambridge, UK), and mouse anti-GAPDH (1:5000, Beyotime Biotechnology, Shanghai, China). Antibodies used for cGAS–STING pathway detection were from the mouse-reactive STING pathway antibody sampler kit (1:1000, Cell Signaling Technology, Danvers, MA, USA). Secondary detection was performed using HRP-conjugated anti-mouse IgG and HRP-conjugated anti-rabbit IgG (1:5000, Beyotime Biotechnology, Shanghai, China).

### 2.9. Construction and Packaging of Lentiviral Particles

The PRTFDC1 overexpression recombinant plasmid pLVX-IRES-ZsGreen1-*PRTFDC1* carrying a 6×His tag and its corresponding control plasmid pLVX-IRES-ZsGreen1-Scramble were constructed by Genewiz (Suzhou, China). A total of 5 × 10^6^ HEK293T cells were seeded in a 10 cm culture dish and cultured for 24 h. When cell confluence reached 80–90%, transfection was performed. The *PRTFDC1* overexpression plasmid or control plasmid was co-transfected with the packaging plasmids psPAX2 and pMD2.G at a mass ratio of 3:2:1. For each 10 cm dish, 10.5 μg, 7 μg, and 3.5 μg of plasmids were used, respectively. Transfection was carried out using PolyJet in vitro DNA transfection reagent (SignaGen Laboratories, Rockville, MD, USA) according to the manufacturer’s protocol, with a transfection reagent-to-DNA ratio of 2:1. After transfection, cells were incubated for 6 h, the medium was replaced with 10 mL of fresh culture medium, and the supernatant was collected after 72 h. The collected supernatant was centrifuged at 5000× *g* for 10 min, filtered through a 0.45 μm membrane, and concentrated using an Amicon Ultra centrifugal filter (100 kDa MWCO; Merck KGaA, Darmstadt, Germany). The concentrated viral suspension was aliquoted into 1.5 mL microcentrifuge tubes and stored at −80 °C.

### 2.10. In Vitro Overexpression and Knockdown of PRTFDC1

To induce PRTFDC1 overexpression in pig MuSCs, cells were infected with a concentrated *PRTFDC1*-overexpressing lentivirus or the corresponding control virus at a multiplicity of infection (MOI) of 0.01 when cell confluence reached approximately 80%. After 48 h of transduction, the medium was replaced with differentiation induction medium, and the cells were further cultured for 2 days. Subsequently, the cells were harvested, and qRT-PCR and Western blot analyses were performed to determine PRTFDC1 mRNA and protein expression levels, thereby confirming the overexpression efficiency.

For PRTFDC1 knockdown, the X-tremeGENE 360 transfection reagent (Merck KGaA, Darmstadt, Germany) was used to transfect pig MuSCs with a specific small interfering RNA (*PRTFDC1*-siRNA-822, sense: 5′-GCAGAUAAUUGGAGGCGAATT-3′; antisense: 5′-UUCGCCUCCAAUUAUCUGCTT-3′; Genecreate, Wuhan, China) targeting *PRTFDC1*. A negative control siRNA (sense: 5′-UUCUCCGAACGUGUCACGUTT-3′; antisense: 5′-ACGUGACACGUUCGGAGAATT-3′) was used as the control. Transfection was carried out simultaneously with the replacement of growth medium by differentiation induction medium, and cells were collected after 4 days of differentiation (DM4). qRT-PCR and Western blot analyses were subsequently performed to assess PRTFDC1 mRNA and protein levels, thereby evaluating the knockdown efficiency.

### 2.11. Statistical Analysis

Data are expressed as the mean ± standard deviation (Mean ± SD). Statistical analyses were performed using SPSS software (v26.0). Group comparisons were conducted with the independent samples *t*-test. A *p*-value < 0.05 was considered statistically significant, *p* < 0.01 was considered highly significant, and *p* < 0.001 was considered extremely significant.

## 3. Results

### 3.1. Differentiation Process and Temporal Regulation of Transcription in Sujiang Pig MuSCs

**Figure 1:**
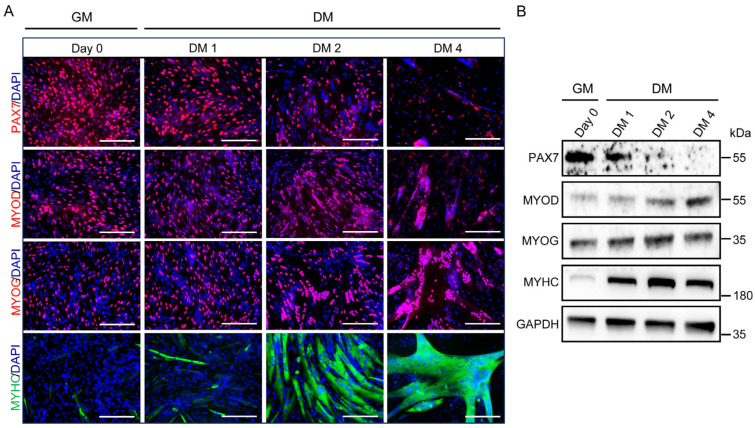
In vitro differentiation process of Sujiang pig skeletal muscle satellite cells (MuSCs). (**A**) Immunofluorescence staining of PAX7, MYOD, MYOG, and MYHC to assess the expression of key markers during the in vitro differentiation of MuSCs. Scale bar = 200 µm. (**B**) Western blotting analysis of the changes in protein expression levels of PAX7, MYOD, MYOG, and MYHC during the in vitro differentiation of pig MuSCs, with GAPDH used as an internal control. Primary antibodies used were mouse anti-PAX7 (1:1000), mouse anti-MYOD (1:1000), mouse anti-MYOG (1:1000), mouse anti-MYHC (1:1000), and mouse anti-GAPDH (1:5000).

MuSCs were isolated from the gastrocnemius muscle of 11-day-old Sujiang pigs using Dispase II and collagenase II for digestion, followed by purification through differential adherence to achieve high-purity MuSCs. Initially, these freshly isolated MuSCs were in a quiescent state. Upon culture in growth medium (GM), they transitioned into myocytes. Once cell confluence reached 90%, differentiation was induced by switching to DM containing 2% FBS. To further investigate the proliferation and differentiation process, cells at various stages—pre-differentiation (GM), 1 day post-differentiation (DM1), 2 days post-differentiation (DM2), and 4 days post-differentiation (DM4)—were analyzed by immunofluorescence and Western blotting. The marker proteins analyzed included PAX7 (a marker of MuSC proliferation), MYOD and MYOG (markers of differentiation), and MYHC (a marker of myotube formation). The results demonstrated that MuSCs in the proliferative phase expressed high levels of PAX7, with the percentage of PAX7-positive cells gradually decreasing as differentiation progressed. MYOD, MYOG, and MYHC, which were expressed at low levels in proliferating MuSCs, were significantly upregulated during differentiation. These findings confirm that Sujiang pig MuSCs were successfully induced to differentiate in vitro (Figure 1).

To explore gene expression changes during MuSC differentiation and identify potential regulators of myogenesis, transcriptome sequencing was performed on samples collected at four stages: GM, DM1, DM2, and DM4. Over 37.12 million clean reads were obtained from each sample, accounting for >99.41% of the raw data, with more than 92.40% of reads uniquely mapped. The Q20 value for all samples was above 98%, and the Q30 value exceeded 94%, indicating high sequencing quality. The GC content ranged from 47% to 48%, reflecting stable read composition (Table S2). To assess mRNA abundance, the distribution of fragments per kilobase of transcript per million mapped reads (FPKM) was analyzed (Figure S1A). A box plot illustrating the gene expression profiles across different stages of differentiation is shown in Figure S1B. Principal component analysis (PCA) clearly distinguished the samples from different stages, revealing significant differences in gene expression profiles between stages (Figure S1C). Clustering analysis based on sample similarity further confirmed that the samples from each differentiation stage clustered appropriately (Figure S1D). These results indicate that the sequencing data were of high quality and reliable.

### 3.2. Analysis of Differentially Expressed Genes (DEGs) During Satellite Cell Differentiation

**Figure 2:**
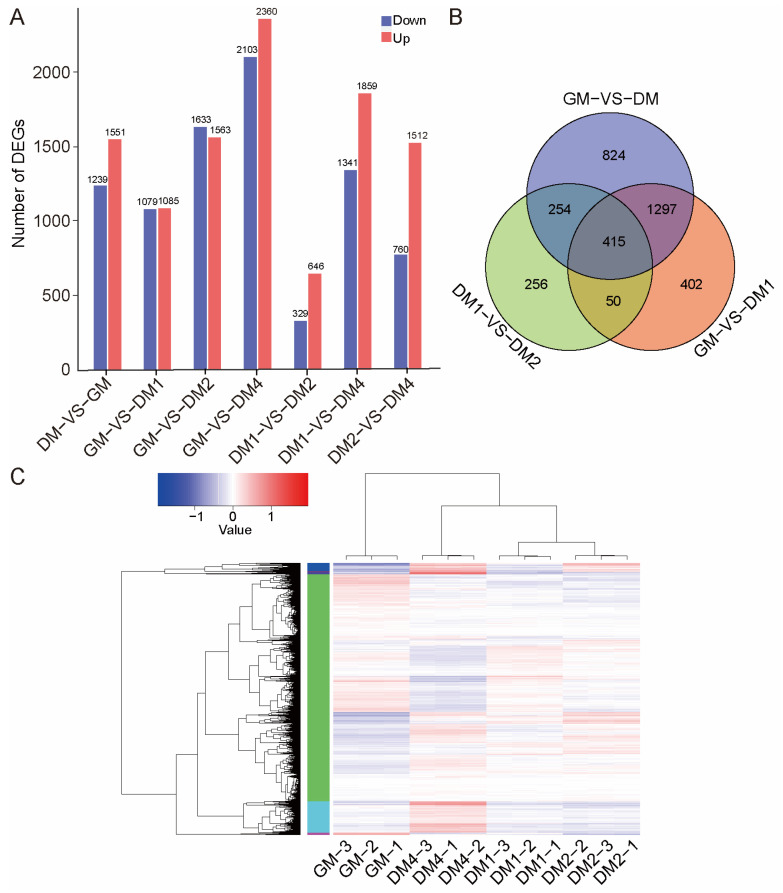
Differentially expressed genes (DEGs) analysis during the differentiation of Sujiang pig MuSCs. (**A**) Upregulation and downregulation of DEGs at four stages of MuSCs differentiation (GM, DM1, DM2, DM4). Upregulated genes are represented by red bars, and downregulated genes are represented by blue bars. (**B**) Venn diagram showing DEGs across different stages. DM refers to pooled DM1, DM2, and DM4 samples. (**C**) Hierarchical clustering heatmap of DEGs, with red representing high expression and blue representing low expression. The color gradient from blue to red indicates increasing gene expression levels. Data represent 3 biological replicates.

DEGs were defined as genes with a fold change ≥ 2 and an Padj ≤ 0.05. A total of 2790 DEGs were identified between pig MuSCs before and after differentiation (DM vs. GM), with 1551 genes upregulated and 1239 genes downregulated (Figure 2A, Table S3). When comparing DM1 to GM, 2164 DEGs were identified, with 1085 genes upregulated and 1079 genes downregulated (Table S4). The comparison between DM2 and DM1 revealed 975 DEGs, including 646 upregulated and 329 downregulated genes (Table S5). The comparison between DM4 and DM2 identified 2272 DEGs, with 1512 upregulated and 760 downregulated genes (Table S6). Hierarchical clustering of the DEGs revealed significant gene expression differences across stages, emphasizing the dynamic transcriptional changes that occur before and during differentiation (Figure 2C). The Venn diagram in Figure 2B highlights the overlap and stage-specific DEGs, with 415 shared genes identified at the intersection. Detailed expression data for these genes are provided in Table S7.

### 3.3. Temporal Expression Patterns of DEGs During Satellite Cell Differentiation

**Figure 3:**
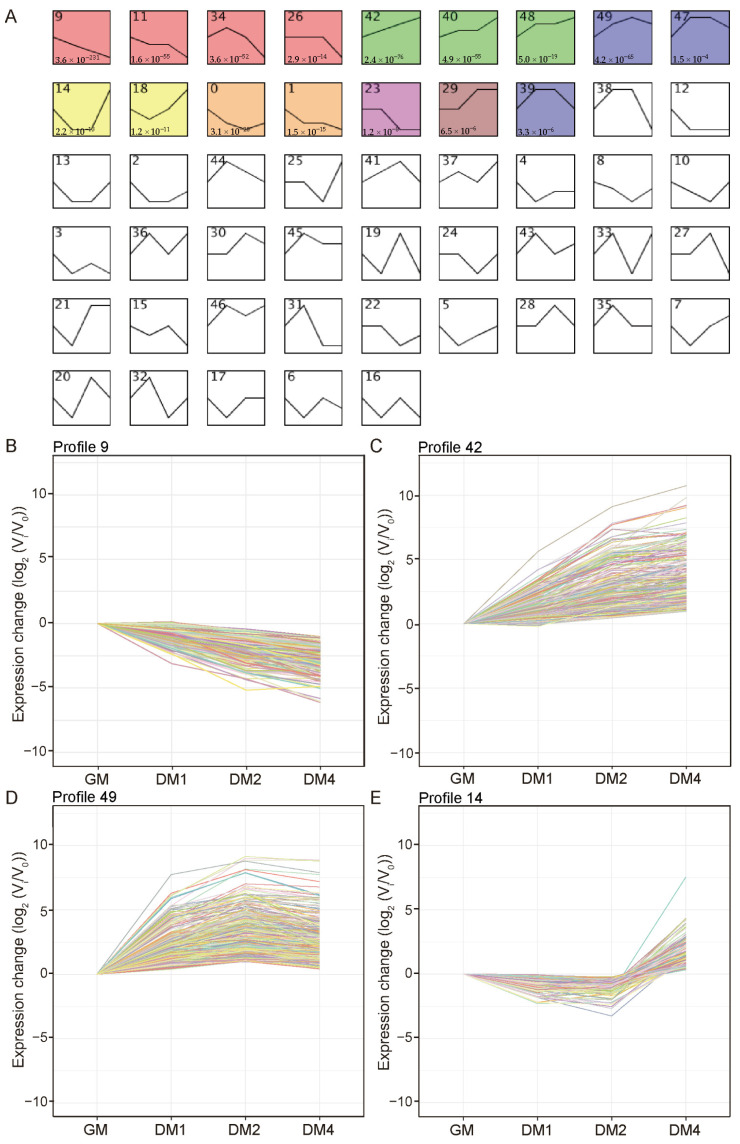
STEM time-series analysis of DEGs during Sujiang pig MuSC differentiation. (**A**) Trend analysis of differentially expressed genes during differentiation. Colors represent statistically significant temporal expression patterns in the clusters. (**B**–**E**) Expression trend plots for genes in profiles 9, 42, 49, and 14. The x-axis represents the samples (GM, DM1, DM2, DM4), and the y-axis shows the log-transformed expression values, with the GM time point set as the control (expression level defined as 0). Other time points are compared to the initial expression levels at the GM time point. Data represent 3 biological replicates.

To characterize distinct expression dynamics during MuSC differentiation, time-series gene expression profiling was performed on GM, DM1, DM2, and DM4 samples using STEM analysis. Sixteen significantly enriched expression profiles were identified, delineating the temporal patterns of gene expression (Figure 3A). These included classical continuously downregulated (Profile 9; containing 620 genes, Figure 3B) and continuously upregulated patterns (Profile 42; containing 373 genes, Figure 3C), as well as more complex spatiotemporal expression modes indicative of intricate regulatory mechanisms. Notably, the up-then-down pattern (Profile 49; Figure 3D) contained 514 genes, such as *RBM24*, *PRKAR1B*, and *TMEM56*, suggesting a potential “switch” role at specific differentiation stages; conversely, the down-then-up pattern (Profile 14; Figure 3E) included 204 genes, such as *ACKR4*, *CAMK4*, and *PPP4R4*, implying their possible involvement in signal pathway remodeling during the late differentiation phase. The complete gene lists and functional annotations are provided in Table S8.

### 3.4. Functional and Pathway Analysis of DEGs Based on GO and KEGG

**Figure 4:**
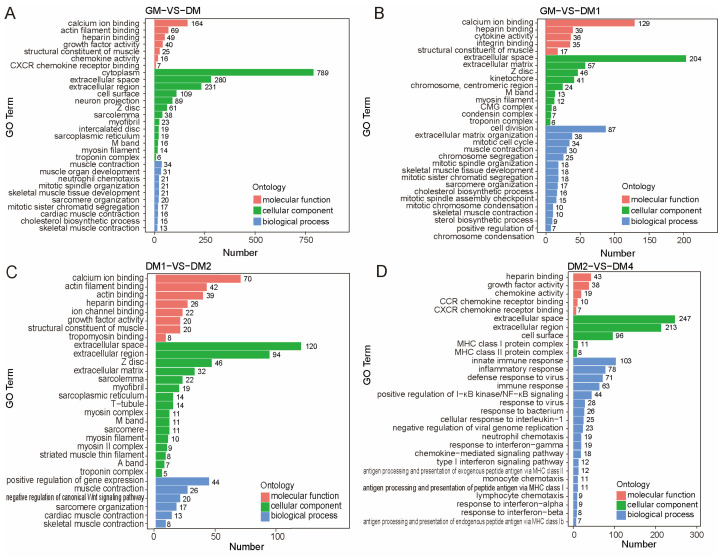
GO analysis of DEGs before and after Sujiang pig MuSC differentiation. (**A**) GO enrichment analysis of DEGs between DM and GM. (**B**) GO enrichment analysis of DEGs between DM1 and GM. (**C**) GO enrichment analysis of DEGs between DM2 and DM1. (**D**) GO enrichment analysis of DEGs between DM4 and DM2. Red represents molecular function, green represents cellular component, and blue represents biological process. The top 30 most significantly enriched GO terms are shown, sorted by GO category and the number of differentially expressed genes. Data represent 3 biological replicates.

To further elucidate the biological roles of the DEGs, GO enrichment analysis was conducted. A total of 971, 882, 896, and 919 significantly enriched GO terms were identified in the DM vs. GM, DM1 vs. GM, DM2 vs. DM1, and DM4 vs. DM2 comparison groups, respectively (Tables S9–S12). The top 30 significantly enriched GO terms for each comparison are illustrated in Figure 4. In the DM vs. GM group, DEGs were mainly enriched in biological processes (BP) associated with muscle development and differentiation, such as muscle contraction, muscle organ development, neutrophil chemotaxis, and skeletal muscle tissue development. At the cellular component (CC) level, DEGs were predominantly localized to muscle-specific structures, including the Z disk, sarcolemma, myofibril, and sarcoplasmic reticulum. Regarding molecular function (MF), they were significantly enriched in calcium ion binding and actin filament binding, collectively delineating a clear functional landscape before and after differentiation (Figure 4A).

**Figure 5:**
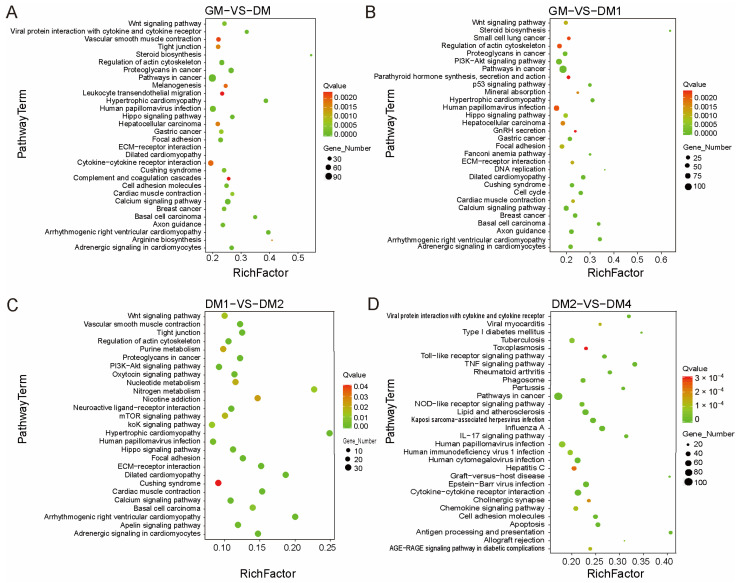
KEGG enrichment scatter plot of DEGs before and after Sujiang pig MuSC differentiation. (**A**–**D**) The top 30 significantly enriched KEGG pathways are shown for DM vs. GM (**A**), DM1 vs. GM (**B**), DM2 vs. DM1 (**C**), and DM4 vs. DM2 (**D**), respectively. The size of each point represents the number of DEGs in each pathway, and the color of the point indicates the corresponding q-value. The top 30 significantly enriched pathways are shown. If fewer than 30 pathways are enriched, all enriched pathways are displayed. Data represent 3 biological replicates.

To explore the signaling pathways potentially involved in muscle differentiation, KEGG pathway enrichment analysis was performed. The results revealed significant enrichment of 81, 69, 27, and 100 pathways in the DM vs. GM, DM1 vs. GM, DM2 vs. DM1, and DM4 vs. DM2 comparison groups, respectively (Tables S13–S16). Figure 5 presents the top 30 significantly enriched pathways for each comparison (or all pathways when fewer than 30). Notably, several well-established signaling pathways known to regulate myogenesis were significantly enriched, including the Wnt, PI3K–Akt, JAK–STAT, p53, Hippo, and Apelin signaling pathways.

### 3.5. Validation of RNA-Seq Results Using qRT-PCR

**Figure 6:**
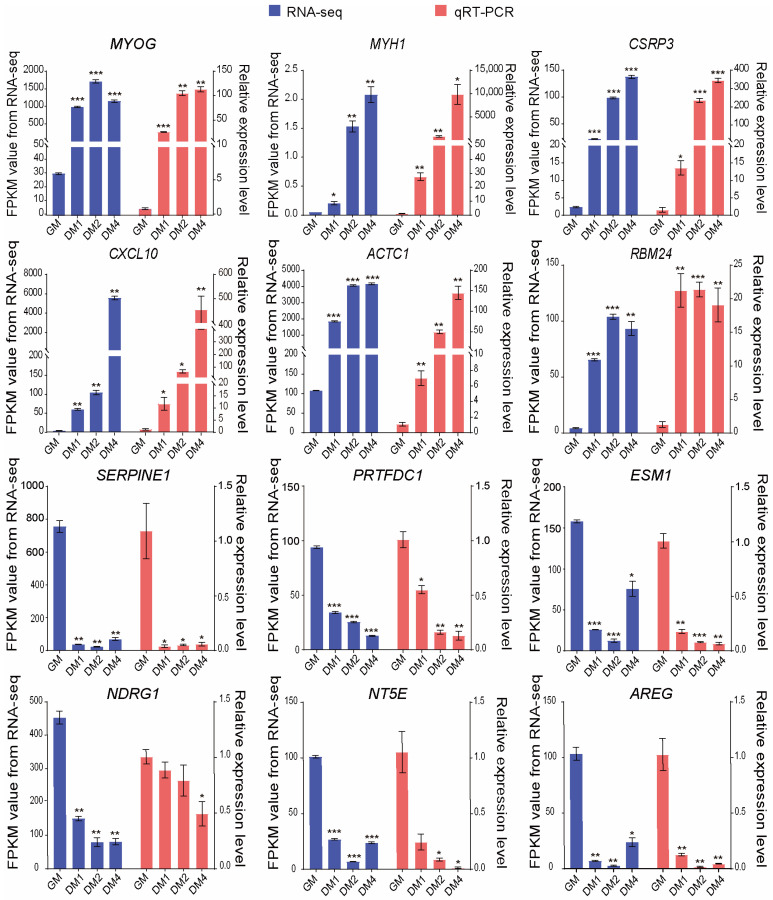
qRT-PCR validation of the expression of 12 DEGs during Sujiang pig MuSC differentiation. The left y-axis represents the FPKM values from RNA-Seq, and the right y-axis represents the qRT-PCR data. The blue bars represent the FPKM values from RNA-Seq, while the pink bars represent the gene expression levels measured by qRT-PCR using 18S rRNA as the reference gene. * indicates *p* < 0.05, ** indicates *p* < 0.01, and *** indicates *p* < 0.001. Data are presented as mean ± SED of 3 biological replicates, each measured in triplicate (technical replicates).

To validate the accuracy and reliability of the RNA-Seq results, qRT-PCR was performed to measure the expression levels of 12 DEGs during differentiation. These included six upregulated genes (*MYOG*, *MYH1*, *CSRP3*, *CXCL10*, *ACTC1*, and *RBM24*) and 6 downregulated genes (*SERPINE1*, *PRTFDC1*, *ESM1*, *NDRG1*, *NT5E*, and *AREG*), with 18S rRNA serving as the reference gene. As illustrated in Figure 6, the expression patterns of these genes were highly consistent with the trends observed in the RNA-Seq data, further confirming the reliability and accuracy of the sequencing results.

### 3.6. Expression Profiling of PRTFDC1 in Sujiang Pig Tissues and During MuSC Differentiation

**Figure 7:**
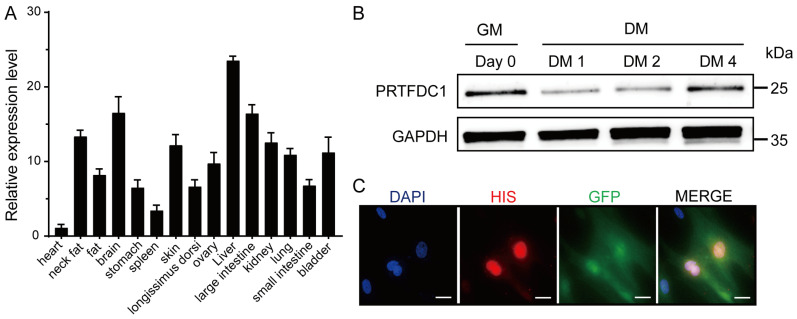
Expression analysis of PRTFDC1 in Sujiang pig tissues and during MuSC differentiation. (**A**) Relative *PRTFDC1* mRNA levels in various Sujiang pig tissues measured by qRT-PCR, with 18S rRNA as the reference gene. Data are presented as mean ± SED of 3 biological replicates, each measured in triplicate (technical replicates). (**B**) Western blot analysis of PRTFDC1 protein expression during in vitro differentiation of pig MuSCs, with GAPDH as the loading control. (**C**) Immunofluorescence showing the subcellular localization of PRTFDC1 in pig MuSCs. The primary antibody was a mouse anti-His tag, and the secondary antibody was goat anti-mouse IgG (H + L) cross-adsorbed secondary antibody, Alexa Fluor 555. Scale bar = 20 μm.

In the DEG analysis described above, *PRTFDC1* was found to be downregulated during pig MuSC differentiation, suggesting that it may serve as a potential regulatory factor in this process. To explore its role, we first examined the tissue expression profile of *PRTFDC1* in 11-day-old Sujiang pigs using qRT-PCR. Using expression in the heart as the reference (set to 1), PRTFDC1 was detected in all tested tissues, showing a ubiquitous expression pattern. Notably, higher expression levels were observed in the liver, brain, and large intestine, while a distinct expression signal was also present in skeletal muscle (Figure 7A). Subsequently, Western blot analysis was performed to assess the protein expression dynamics of PRTFDC1 during MuSC differentiation. The results revealed a marked reduction in PRTFDC1 protein abundance at the early differentiation stage (Figure 7B), consistent with transcriptomic and qRT-PCR findings. Interestingly, PRTFDC1 protein levels showed a slight rebound at the late differentiation stage, implying that it may play stage-specific regulatory roles during MuSC differentiation. Although PRTFDC1 is endogenously downregulated during differentiation, exogenous overexpression promotes MuSC differentiation, representing an apparent “inverse relationship” that is further discussed later. To further determine its subcellular localization, a 6×His-tagged *PRTFDC1* overexpression construct (pLVX-IRES-ZsGreen1-*PRTFDC1*) was generated and introduced into MuSCs via a lentiviral transduction system. Immunofluorescence analysis using an anti-His antibody revealed that the PRTFDC1 protein was predominantly localized in the nucleus (Figure 7C).

### 3.7. Effects of PRTFDC1 Overexpression and Knockdown on the Differentiation of Sujiang Pig MuSCs

**Figure 8:**
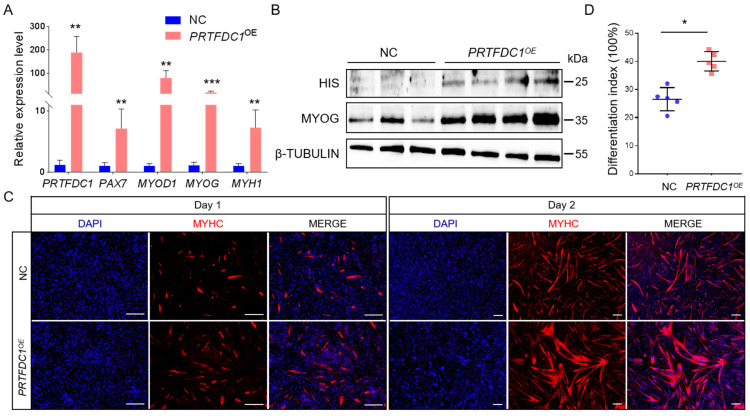
Overexpression of *PRTFDC1* (*PRTFDC1^OE^*) promotes MuSC differentiation into myotubes. (**A**) qRT-PCR analysis of relative mRNA expression levels of *PRTFDC1*, *PAX7*, *MYOD1*, *MYOG*, and *MYH1* in *PRTFDC1^OE^* MuSCs after 2 days of induced differentiation. 18S rRNA was used as the reference gene. Cells infected with PLVX-IRES-ZsGreen1-Scramble lentivirus served as the negative control. Data are presented as mean ± SED of 6 biological replicates, each measured in triplicate (technical replicates). (**B**) Western blot analysis of PRTFDC1 and MYOG protein expression in *PRTFDC1^OE^* MuSCs after 2 days of induced differentiation, with β-TUBULIN as the loading control. Primary antibodies: mouse anti-His tag (1:1000) for PRTFDC1 and rabbit anti-β-TUBULIN (1:5000). (**C**) Immunofluorescence staining of MYHC showing myotube formation in *PRTFDC1^OE^* MuSCs at 1 and 2 days of induced differentiation. Scale bar = 200 μm. (**D**) Quantification of the differentiation index, calculated as the percentage of nuclei within myotubes containing ≥2 nuclei relative to total nuclei. Data are presented as mean ± SED of 5 biological replicates from day 2 of induced differentiation. * *p* < 0.05, ** *p* < 0.01, *** *p* < 0.001.

To elucidate the functional role of PRTFDC1 during Sujiang pig MuSC differentiation, both overexpression and knockdown experiments were performed. A lentiviral vector carrying the *PRTFDC1* coding sequence (pLVX-IRES-ZsGreen1-*PRTFDC1*, containing a C-terminal 6×His tag) was constructed and packaged, while a control lentivirus harboring a nonspecific sequence (pLVX-IRES-ZsGreen1-Scramble) served as the negative control. After 48 h of infection, the culture medium was replaced with differentiation induction medium. MYHC immunofluorescence staining was used to assess myotube formation. Two days after induction, the *PRTFDC1*-overexpressing group (*PRTFDC1^OE^*) exhibited a pronounced increase in myotube number, with longer and more extensively fused myotubes compared with the control group (Figure 8C). Quantitative analysis revealed a significantly higher differentiation index (ratio of nuclei within myotubes containing ≥2 nuclei to total nuclei; 26.5% vs. 40.1%, *p* < 0.05; Figure 8D). qRT-PCR results showed a marked upregulation of *PRTFDC1* mRNA expression (*p* < 0.01), along with increased transcript levels of myogenic marker genes *MYOD1*, *MYOG*, and *MYH1* (Figure 8A). Western blot analysis confirmed these findings, demonstrating that PRTFDC1 overexpression markedly enhanced MYOG protein expression (Figure 8B).

**Figure 9:**
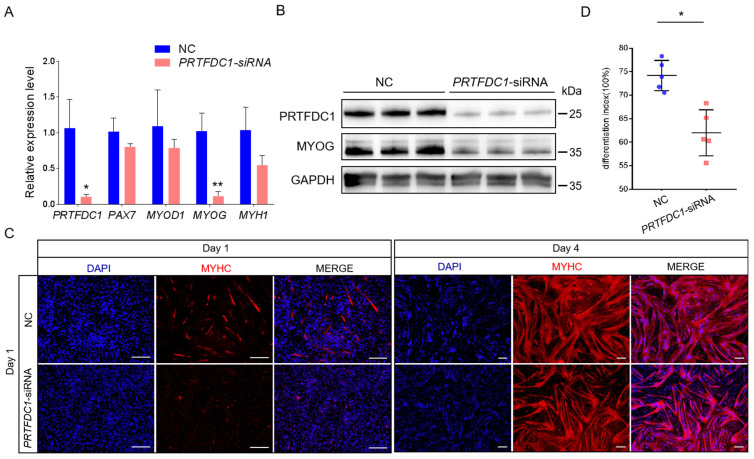
Knockdown of *PRTFDC1* (*PRTFDC1-siRNA*) inhibits MuSC differentiation into myotubes. (**A**) qRT-PCR analysis of relative mRNA expression levels of *PRTFDC1*, *PAX7*, *MYOD1*, *MYOG*, and *MYH1* in *PRTFDC1-siRNA* MuSCs after 4 days of induced differentiation. 18S rRNA was used as the reference gene, and non-targeting siRNA served as the negative control. Data are presented as mean ± SED of 4 biological replicates, each measured in triplicate (technical replicates). (**B**) Western blot analysis of PRTFDC1 and MYOG protein expression in *PRTFDC1-siRNA* MuSCs after 4 days of induced differentiation, with GAPDH as the loading control. Primary antibodies: rabbit anti-PRTFDC1 (1:1000) and mouse anti-GAPDH (1:5000). (**C**) Immunofluorescence staining of MYHC showing myotube formation in *PRTFDC1-siRNA* MuSCs at 1 and 4 days of induced differentiation. Scale bar = 200 μm. (**D**) Quantification of the differentiation index in cells after 4 days of induced differentiation. Data are presented as mean ± SED of 5 biological replicates. * *p* < 0.05, ** *p* < 0.01.

To further validate the role of PRTFDC1, a specific siRNA targeting *PRTFDC1* (*PRTFDC1*-siRNA-822) was designed and synthesized, with a non-targeting siRNA used as the negative control. The siRNA was transfected simultaneously with the induction of differentiation. After 4 days of differentiation, myotube formation was markedly inhibited in the *PRTFDC1*-knockdown group, and the differentiation index significantly decreased (74.0% vs. 61.3%, *p* < 0.05; Figure 9C,D). Both qRT-PCR and Western blot analyses confirmed a significant reduction in PRTFDC1 expression (*p* < 0.05), accompanied by a marked decrease in MYOG expression (Figure 9A,B).

Collectively, these results demonstrate that PRTFDC1 overexpression promotes myotube formation, whereas its knockdown inhibits differentiation, indicating that PRTFDC1 acts as a positive regulator of pig MuSC differentiation.

### 3.8. Effects of PRTFDC1 on the cGAS–STING Signaling Pathway

**Figure 10:**
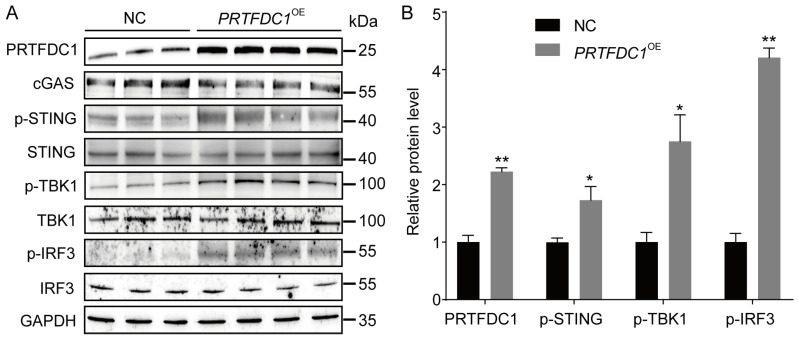
Overexpression of PRTFDC1 activates the cGAS–STING signaling pathway in Sujiang pig MuSCs. (**A**) Western blot analysis showing total and phosphorylated protein levels of cGAS, STING, TBK1, and IRF3 in MuSCs following PRTFDC1 overexpression. GAPDH was used as the loading control. (**B**) Densitometric quantification of protein bands. Data are presented as mean ± SED of 3 biological replicates, each measured in triplicate (technical replicates). * *p* < 0.05, ** *p* < 0.01.

Given that PRTFDC1 regulates the differentiation of pig MuSCs, we further investigated its potential molecular mechanism. Previous studies have demonstrated that the cGAS–STING signaling pathway, an intracellular DNA-sensing system, is involved not only in immune responses but also in cellular processes such as proliferation, apoptosis, and differentiation [24,25,26]. Moreover, recent evidence suggests that the cGAS–STING pathway functions as a novel regulatory mechanism in myogenic differentiation [27,28]. To determine whether PRTFDC1 exerts its effects through this pathway, we analyzed the protein expression of cGAS, STING, and their downstream signaling molecules TBK1 and IRF3 in *PRTFDC1^OE^* MuSCs using Western blotting. The results showed that PRTFDC1 overexpression markedly increased the phosphorylation levels of STING, TBK1, and IRF3 (Figure 10A,B). These findings indicate that the regulatory role of PRTFDC1 may be associated with the cGAS–STING signaling pathway.

## 4. Discussion

The growth and development of skeletal muscle depend on the proliferation and differentiation of satellite cells. Elucidating the regulatory mechanisms governing their differentiation not only clarifies the molecular basis of muscle development but also provides a theoretical foundation for improving meat quality traits in pigs. In this study, MuSCs were isolated from three Sujiang pigs, and an in vitro differentiation system was established. Temporal transcriptomic changes during differentiation were systematically analyzed using mRNA sequencing, revealing key regulatory genes and signaling pathways with significant dynamic changes. Notably, PRTFDC1 expression decreased markedly during differentiation, and functional assays demonstrated that its overexpression promoted myotube formation, whereas knockdown inhibited differentiation, indicating a positive regulatory role in MuSC differentiation. Furthermore, PRTFDC1 enhanced the phosphorylation of key proteins in the cGAS–STING signaling pathway. Collectively, these findings provide novel transcriptomic resources for understanding the dynamic regulatory network underlying porcine skeletal muscle development and highlight potential target genes for molecular breeding aimed at enhancing muscle growth.

Through transcriptome sequencing, this study characterized the dynamic gene expression patterns of Sujiang pig MuSCs during the proliferation and successive differentiation stages. Consistent with previous findings, classical myogenic marker genes such as *MYOD1*, *MYOG*, *MYOMAKER*, and *MYH1* were significantly upregulated during differentiation. Several genes previously reported to be associated with muscle growth and satellite cell differentiation were also identified, including *DDIT4L*, *XIRP2*, *NES*, and *TRHR*. According to the literature, *DDIT4L* acts as a negative regulator of the mTOR signaling pathway and modulates muscle growth and metabolism by inhibiting mTOR activity [29,30]. *XIRP2* binds to actin filaments and participates in cytoskeletal assembly [31]. *NES*, an intermediate filament protein, is believed to play an essential role in maintaining the structural integrity of MuSCs and promoting muscle differentiation [32,33]. Polymorphisms in *TRHR* have been associated with sarcopenia, an age-related disorder characterized by muscle atrophy and functional decline, although its specific regulatory mechanism remains unclear [34,35]. The identification of these genes supports the reliability and comprehensiveness of the transcriptomic data obtained in this study. In addition, several novel candidate genes potentially involved in MuSC differentiation were discovered, such as *FRMD4B*, *LRIT1*, and *FLVCR2*. Although these genes have not been previously reported in relation to satellite cell differentiation, their low expression during proliferation and significant upregulation during differentiation suggest potential functional roles in myogenic regulation, warranting further investigation.

To capture key transition points from proliferation to differentiation and to reveal dynamic gene expression changes during myogenesis, cell samples were collected at four representative time points (GM, DM1, DM2, and DM4) for transcriptome sequencing. This sampling strategy enabled a comprehensive depiction of the molecular characteristics of MuSCs at distinct differentiation stages. As shown in Figure 1A, on day 1 of differentiation, cells began to fuse and form nascent myotubes. Compared with the proliferation phase, this early differentiation stage is critical for identifying the key regulators initiating differentiation. On day 2, extensive cell fusion occurred, and both the number and diameter of myotubes increased markedly, making this stage suitable for identifying core genes that promote myotube formation. By analyzing gene expression across these time points, we system MuSCs from proliferation to differentiation. Time-series expression analysis identified 16 significantly enriched expression profiles (Figure 3; Table S8) with diverse temporal expression patterns. In addition to continuously upregulated or downregulated profiles, several exhibited distinct stage-specific dynamics. For example, some genes exhibited an “up–then–down” pattern, such as Profiles 34, 42, and 39, with expression peaking at early differentiation and gradually decreasing thereafter, suggesting their involvement in initiating differentiation. Another group displayed a “down–then–up” pattern, such as Profiles 14 and 18, including *ACKR4*, *CAMK4*, and *PPP4R4*, which were suppressed during early differentiation but recovered or increased at later stages, implying roles in the maturation or maintenance of differentiated cells. These temporally dynamic changes reflect participation in complex regulatory networks that warrant further functional investigation.

To elucidate the molecular mechanisms through which DEGs regulate MuSC differentiation and muscle development, GO annotation and KEGG pathway enrichment analyses were performed for the four comparison groups (DM vs. GM, DM1 vs. GM, DM2 vs. DM1, and DM4 vs. DM2). DEGs were significantly enriched in biological processes and molecular functions related to muscle structure and function, including muscle contraction, muscle organ development, neutrophil chemotaxis, skeletal muscle tissue development, calcium ion binding, and actin filament binding. KEGG enrichment analysis identified Wnt, PI3K–Akt, JAK–STAT, Hippo, p53, and Apelin signaling pathways in our dataset. These pathways are well-established regulators of myogenesis (Wnt [36,37,38,39,40], PI3K–Akt [41,42,43], JAK–STAT [44,45], Hippo [46], p53 [47,48,49], and Apelin [28,50]). These results are consistent with previous studies on MuSC differentiation and myogenesis, confirming the robustness of our transcriptome data. Overall, pig MuSCs activate a series of muscle formation–related signaling pathways during differentiation, highlighting the essential roles in regulating MuSC fate determination and myofiber maturation. This provides a theoretical basis for dissecting the specific mechanisms of key genes and signaling pathways involved in skeletal muscle development and regeneration.

During DEG screening, we observed that *PRTFDC1* exhibited significant differential expression before and after differentiation. The *PRTFDC1* gene encodes a protein containing a phosphoribosyl transferase domain and belongs to the purine/pyrimidine phosphoribosyl transferase family. Its structure shows high similarity to that of the classical catalytic enzyme hypoxanthine-guanine phosphoribosyltransferase (HPRT) and is highly conserved among mammals [51,52]. Although PRTFDC1 possesses a phosphoribosyl transferase-like domain, studies have suggested that it may lack typical catalytic activity and instead function as a regulatory factor or pseudoenzyme involved in protein–protein interactions and signaling pathway regulation [53]. Previous research has demonstrated that PRTFDC1 is closely associated with various neuropsychiatric and stress-related disorders, such as dissociative amnesia and acute stress reaction [54,55], and plays important roles in the occurrence and progression of multiple cancers [56,57,58]. However, its role in skeletal muscle growth remains largely unexplored. In this study, the transcriptional level of *PRTFDC1*, determined by RNA-seq and qRT-PCR, exhibited a consistent downward trend during MuSC differentiation, whereas its protein abundance increased again at the late differentiation stage (DM4). This discrepancy between mRNA and protein expression suggests that PRTFDC1 may be subject to multilayered regulation, including post-translational modifications, altered protein stability, or changes in translation efficiency. Future investigations such as protein half-life tracking and proteasome or autophagy inhibition assays could help determine whether the observed increase in protein abundance arises from enhanced stability or improved translational control.

Interestingly, although endogenous PRTFDC1 expression decreased during differentiation, its exogenous overexpression markedly promoted myotube formation, characterized by larger myotubes, a higher differentiation index, and increased expression of key myogenic markers *MYOD1*, *MYOG*, and *MYH1*. Conversely, PRTFDC1 knockdown inhibited differentiation. This inverse relationship between endogenous expression and phenotypic outcome suggests that PRTFDC1 function may depend on stage-specific activity thresholds and interaction networks. In other words, its spatiotemporal expression, subcellular localization, and post-translational modifications (e.g., phosphorylation or ubiquitination) may determine distinct regulatory effects on downstream signaling pathways such as cGAS–STING. Future stage-specific or dose-dependent perturbation experiments will be valuable for elucidating the temporal dynamics of PRTFDC1 function.

Previous studies have demonstrated that the cGAS–STING signaling pathway, beyond its established role in innate immunity, also regulates cell proliferation, apoptosis, autophagy, and differentiation [24,25,59,60]. Our findings indicate that PRTFDC1 overexpression enhances the phosphorylation levels of STING and its downstream effectors TBK1 and IRF3, suggesting that it may promote pig MuSC differentiation through activation of the cGAS–STING pathway. However, the precise molecular mechanisms remain to be clarified. Possible regulatory models include PRTFDC1 acting as a nuclear regulator that directly or indirectly promotes the transcription of cGAS–STING-related genes, influencing intracellular DNA homeostasis by facilitating the release of cytosolic or mitochondrial DNA to activate cGAS, or forming a complex with STING to enhance its stability and promote signal transduction. Future Co-IP or IP–MS experiments could determine whether PRTFDC1 physically interacts with STING, while cytoplasmic DNA quantification and cGAS/STING interference assays using siRNA or small-molecule inhibitors could help clarify its hierarchical role within this pathway.

Collectively, PRTFDC1 exhibits a complex, stage-dependent regulatory pattern during pig MuSC differentiation. The downregulation of its endogenous expression does not necessarily indicate an inhibitory role; rather, moderate expression or localized activation of PRTFDC1 may facilitate myotube formation and differentiation by modulating the cGAS–STING signaling pathway. This study has certain limitations, and future in-depth investigations into the temporal expression, dosage effects, and molecular mechanisms of PRTFDC1 will help clarify its critical role in skeletal muscle development and regeneration, providing new theoretical insights and potential molecular targets for improving muscle traits in pigs and regulating muscle regeneration.

## 5. Conclusions

Our study offers valuable insights into the molecular regulation of skeletal muscle development in pigs. In summary, this study systematically characterized the temporal transcriptomic dynamics of Sujiang pig MuSCs during proliferation and successive differentiation stages, providing an integrated view of the molecular networks governing myogenesis. PRTFDC1 was identified as a novel positive regulator potentially involved in skeletal muscle differentiation, with preliminary evidence suggesting it may modulate this process via the cGAS–STING signaling pathway. These findings advance our understanding of the molecular mechanisms underlying MuSC differentiation and establish a theoretical basis for future applications in muscle regeneration and genetic improvement in pigs.

## Data Availability

The data presented in this study are openly available in [NCBI] [https://www.ncbi.nlm.nih.gov/bioproject/PRJNA1224549/; accessed on 17 February 2025] [PRJNA1224549].

## Supplementary Materials

The following supporting information can be downloaded at https://www.mdpi.com/article/10.3390/vetsci12121197/s1. Tables S1 and S2 and Figure S1 are provided in the supplementary data file. Table S1: Primer sequences for qRT-PCR validation. Table S2: Summary of the RNA-Seq data for each sample. Table S3: Group_DM-VS-GM_DE_significant_anno. Table S4: Group_DM1-VS-GM_DE_significant_anno. Table S5: Group_DM2-VS-DM1_DE_significant_anno. Table S6: Group_DM4-VS-DM2_DE_significant_anno. Table S7: Venn_table_anno. Table S8: Expression Trend Analysis. Table S9: GO_DM-VS-GM_significant. Table S10: GO_DM1-VS-GM_significant. Table S11: GO_DM2-VS-DM1_significant. Table S12: GO_DM4-VS-DM2_significant. Table S13: KEGG_DM-VS-GM_significant. Table S14: KEGG_DM1-VS-GM_significant. Table S15: KEGG_DM2-VS-DM1_significant. Table S16: KEGG_DM4-VS-DM2_significant. Figure S1: mRNA expression analysis. Figure S2: Original image of Figure 1B. Figure S3: Original image of Figure 7B. Figure S4: Original image of Figure 8B. Figure S5: Original image of Figure 9B. Figure S6: Original image of Figure 10A.

## Abbreviations

The following abbreviations are used in this manuscript:

MuSCs	Skeletal muscle satellite cells
DEGs	Differentially expressed genes
mRNA-Seq	mRNA sequencing
GO	Gene Ontology
KEGG	Kyoto Encyclopedia of Genes and Genomes
PCA	Principal component analysis
BP	Biological process
CC	Cellular component
MF	Molecular function
