# Supplementary material for: Transcriptomic Insights Reveal PRTFDC1 as a Novel Regulator of Myogenic Differentiation in Sujiang Pig Satellite Cells
Source: Vet Sci. 2025 Dec 14;12(12):1197. doi: 10.3390/vetsci12121197 (PMC12737788; doi:10.3390/vetsci12121197)
Supplement: Supplementary file 1 [file vetsci-12-01197-s001.zip › vetsci-4002710 Supplementary data.pdf]

Supplemental Information

Transcriptomic Insights Reveal PRTFDC1 as a Novel Regulator of Myogenic Differentiation in Sujiang Pig Satellite Cells

Li Zhang[1,2], Xiaowei Ye[2], Suyi Sun[2], Lei Zhang[4], Yixin Gu[2], Shinuo Cao[1,2], Mo Zhou[1,2], Weixiang Sun[1,2], Changyao Fu[2], Qingqing Zhang[2], Mei Li[2], Ziyue Xu[2], Wei Miao[2], Qinse Xu[3*], Shanyuan Zhu[1,2*]

Table S1. Primer sequences for qRT-PCR validation.

Gene	Primer Sequence(5′-3′)	Product Size/bp	NCBI Gene ID
MYOG	F: AGTGAATGCAGTTCCCACAG<br>R: GAGGTGAGGGAGTGCAGATT	131	497618
MYH1	F: TGAGGAAGCGGAGGAACAAT<br>R: TGAACCTCCCGACTCTTGAC	138	100125538
CSRP3	F: GCGACAAGACCGTCTACCAC<br>R: TGACTCGTGAGCTGCTACCG	125	100337687
CXCL10	F: CTGTTCGCTGTACCTGCATC<br>R: TGATCTCAACATGTGGGCAAG	108	494019
ACTC1	F: GTCATGGTGGGTATGGGTCA<br>R: CCTCAGTGAGCAGAGTAGGG	196	100152267
RBM24	F: CGGGGCTATGGATTTGTCAC<br>R: CCTTCCTGCCGTCAATGATG	88	100156013
SERPINE1	F: CTCCTCCCATCACCAGTCTC<br>R: TTGAACTGCATGGCCTCTTG	195	396945
PRTFDC1	F: TTGAACGGCTGGCTAAGGAT<br>R: CTTTCAGCACACACAGGACC	72	100518211
ESM1	F: GCATGACATGGCATCTGGAG<br>R: ACTTCATTCCAGGAGACCGG	83	100739671
NDRG1	F: TCCTGCTTTGTTGGTGGTTG<br>R: CCACAATCCGCCATCTTGAG	111	100156071
NT5E	F: GCAACCCACCTTCCAAAGAG<br>R: ACTCCACCTTCAAGTAGCCC	129	100157995
AREG	F: GCTGCTTTTGTCTCTGCCAT<br>R: TTTCGTTCTTCAGCTGCACC	107	397668
MYOD1	F: TTCGAGACTCTCAAGCGCTG<br>R: TGCAGGCCTTCGATATAGCG	98	407604
PAX7	F: GGTGGGGTCTTCATCAATGG<br>R: GTCTCTTGGTAGCGGCAGAG	155	100625823
18S rRNA	F: CCTGCGGCTTAATTTGACTC<br>R: ATGCCAGAGTCTCGTTCGTT	168	/

Table S2. Summary of the RNA-Seq data for each sample.

Sample	Raw Reads	Clean Reads	Uniquely mapped	Clean Q20 (%)	Clean Q30 (%)	Clean GC (%)
GM-1	43465598	43209202	40171704 (92.9703%)	98.07	94.54	47.99
GM-2	46187054	45957416	42755954 (93.0339%)	98.14	94.70	48.28
GM-3	37296644	37123452	34681743 (93.4227%)	98.20	94.85	47.95
DM1-1	40033522	39866252	36990403 (92.7863%)	98.18	94.80	48.50
DM1-2	41787796	41578322	38618225 (92.8807%)	98.22	94.90	48.68
DM1-3	44685002	44466246	41394025 (93.0909%)	98.24	94.97	48.59
DM2-1	41463520	41245274	38604458 (93.5973%)	98.20	94.89	48.09
DM2-2	38850740	38654426	35828261 (92.6886%)	98.16	94.75	48.22
DM2-3	53064412	52789386	48869802 (92.5751%)	98.26	95.01	47.85
DM4-1	38290304	38067822	35173255 (92.3963%)	98.07	94.55	47.31
DM4-2	41303760	41119254	38016449 (92.4541%)	98.21	94.89	47.85
DM4-3	39556992	39384226	36514004 (92.7123%)	98.32	95.16	47.63

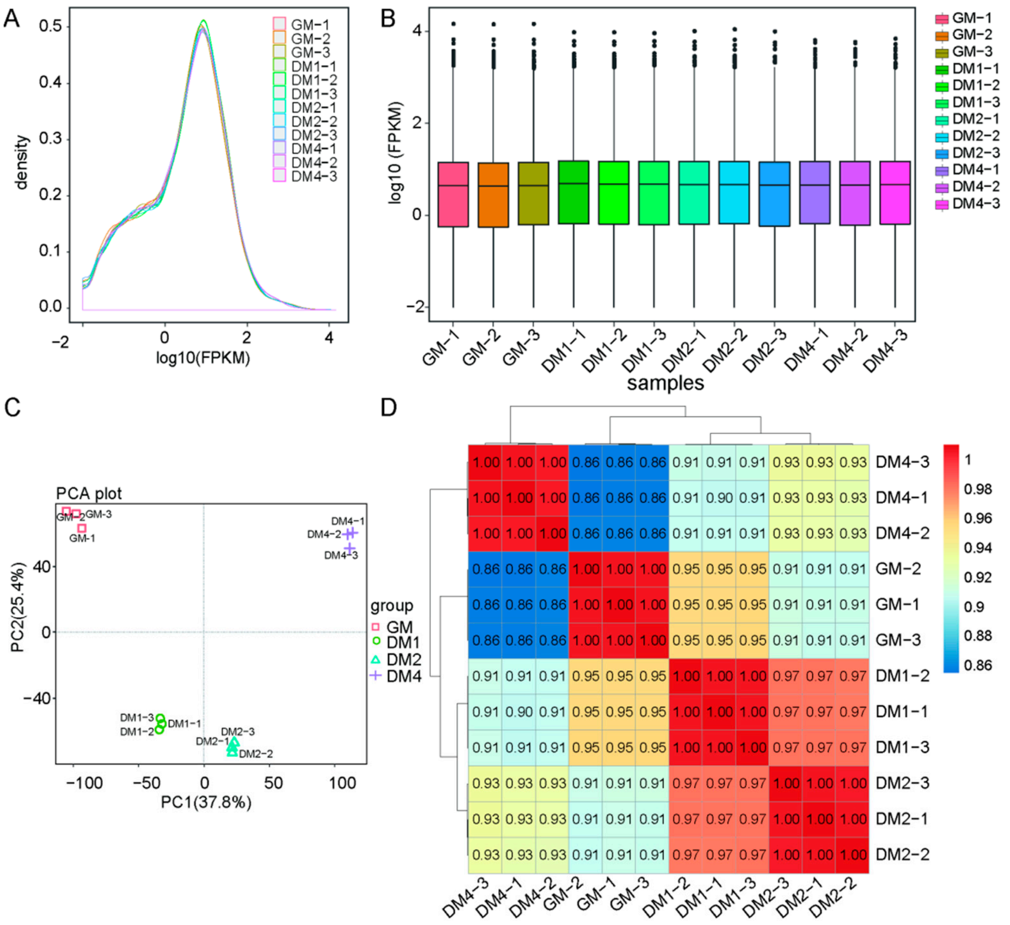

Figure S1. mRNA expression analysis. (A) Density distribution of mRNAs based on log10(FPKM). (B) Box plot of FPKM values for 12 samples (GM-1, GM-2, GM-3, DM1-1, DM1-2, DM1-3, DM2-1, DM2-2, DM2-3, DM4-1, DM4-2, DM4-3), with the y-axis representing log10(FPKM). The box plot displays the maximum value, upper quartile, median, lower quartile, and minimum value. (C) Principal component analysis (PCA) of the 12 samples. (D) Hierarchical clustering analysis of the 12 samples.

Figure S2: Original image of Figure 1b

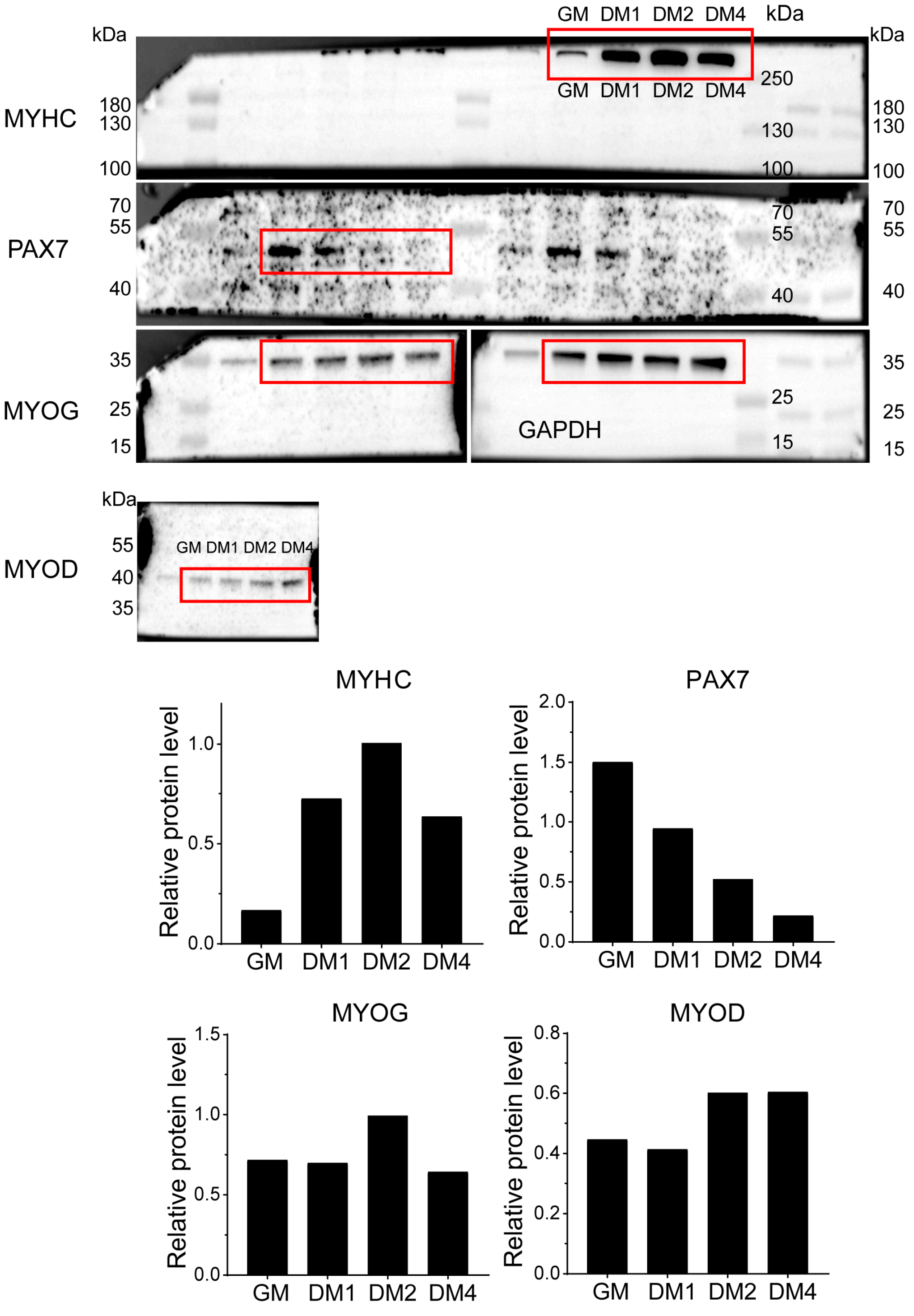

Figure S3: Original image of Figure 7b

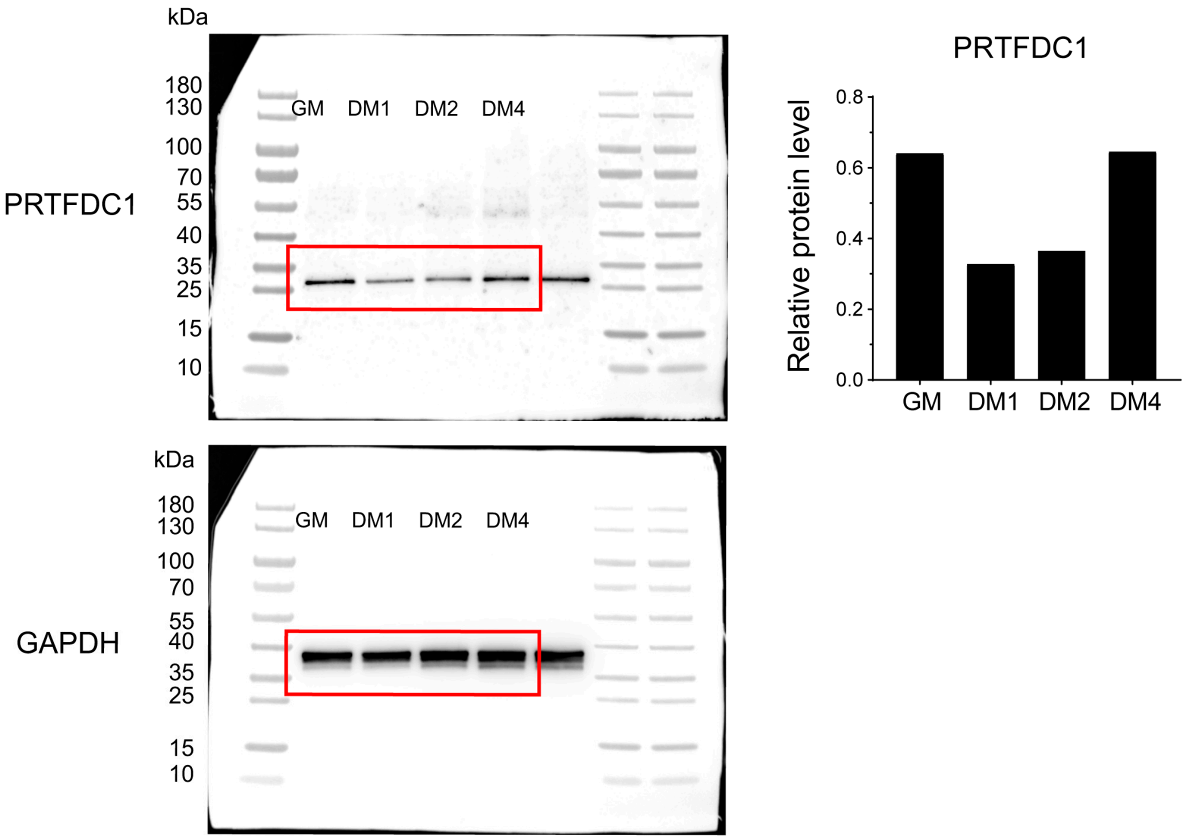

Figure S4: Original image of Figure 8b

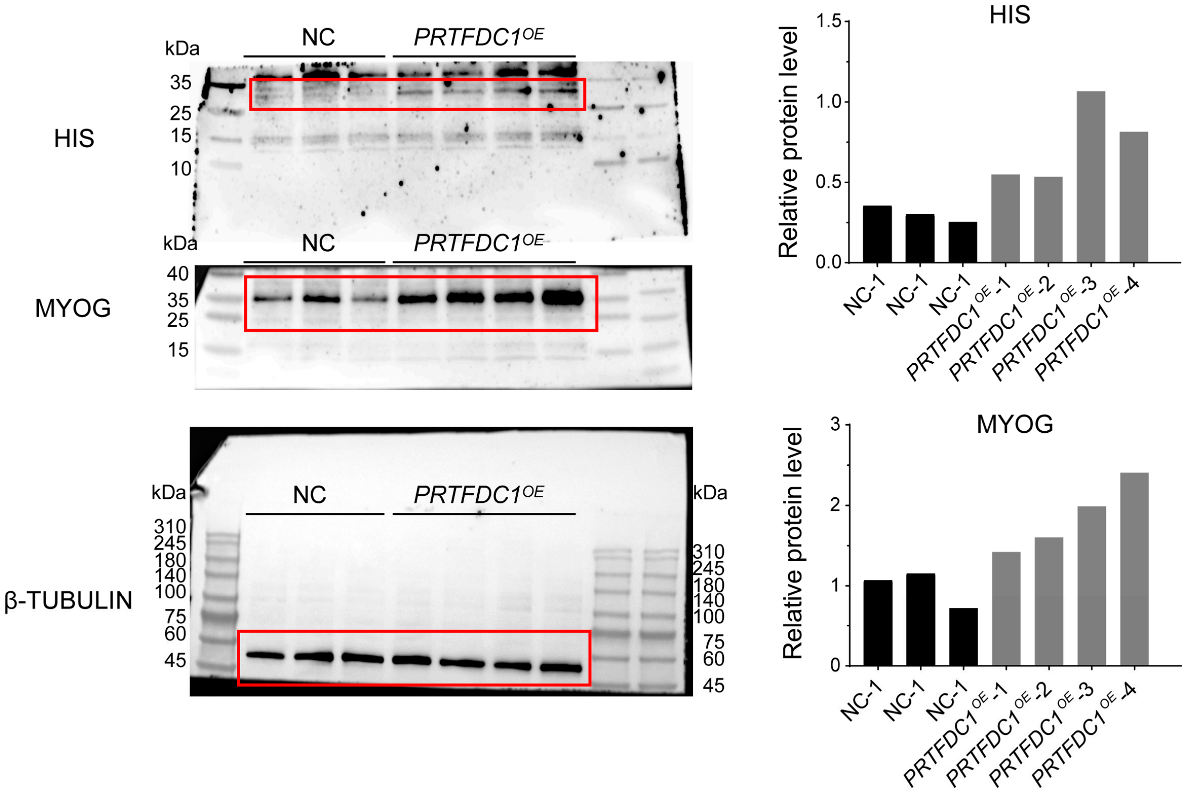

Figure S5: Original image of Figure 9b

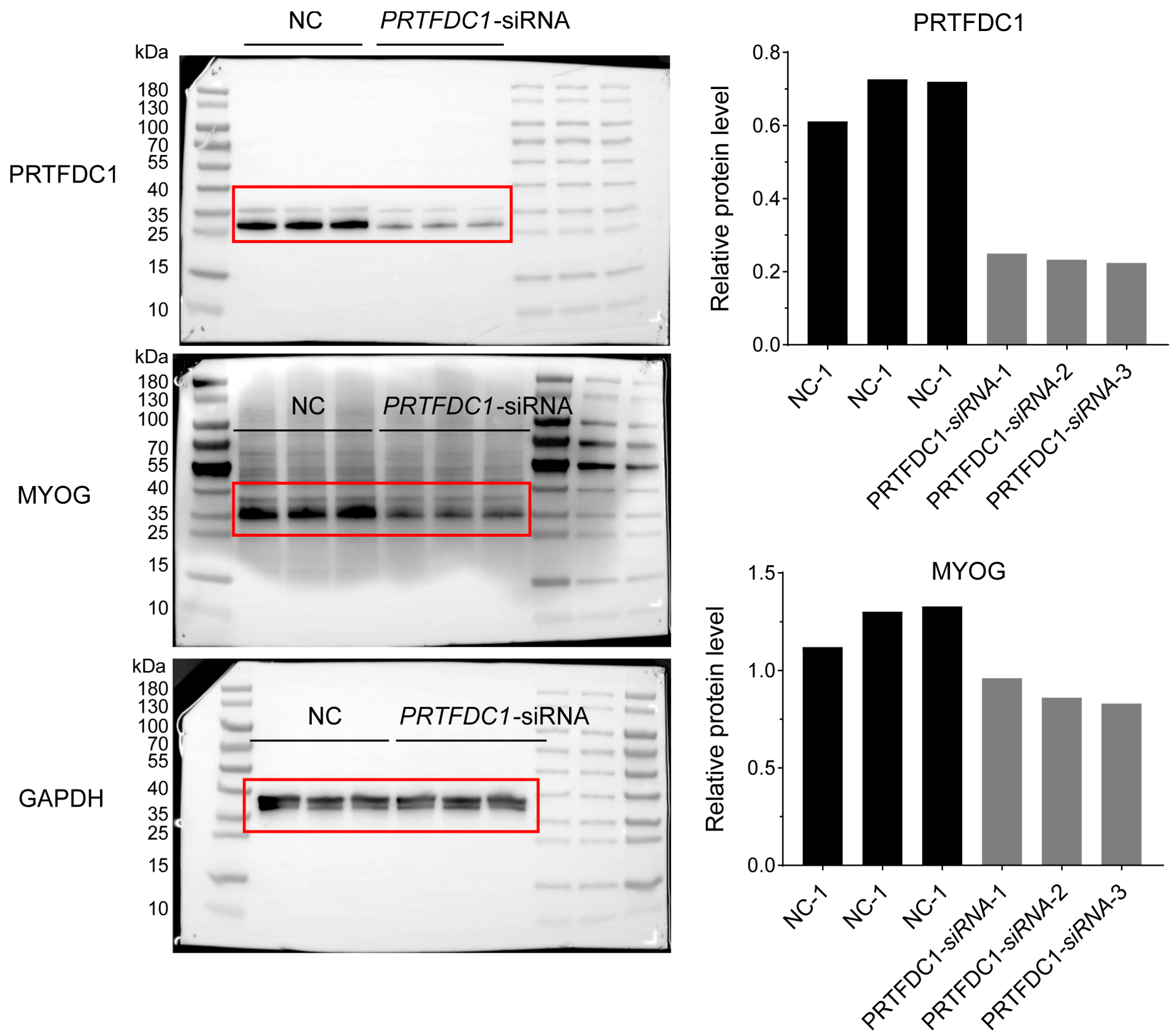

Figure S6: Original image of Figure 10a

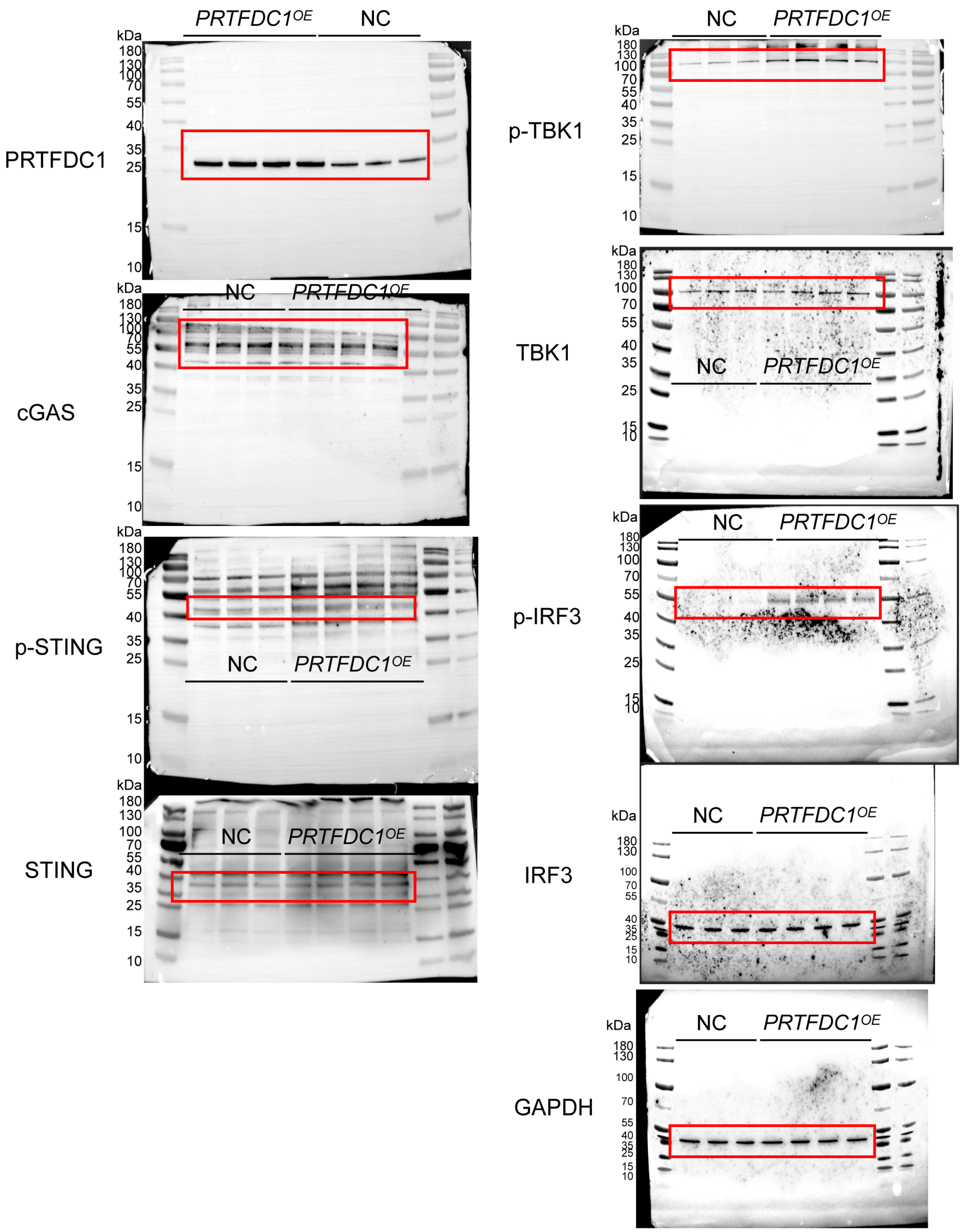

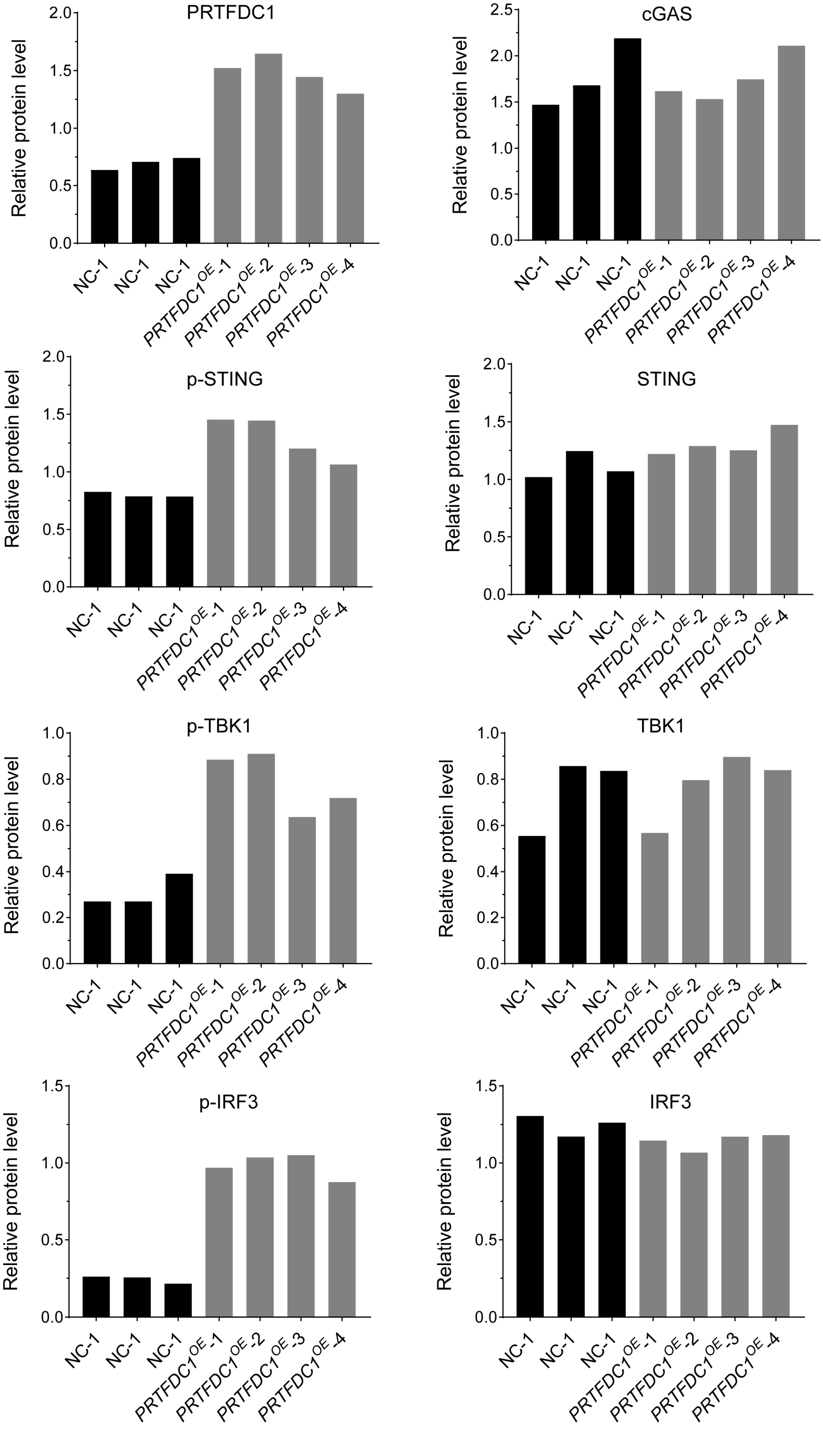
PRTFDC1
cGAS
p-STING
STING
p-TBK1
TBK1
p-IRF3
IRF3
Relative protein level
0.0
0.2
0.4
0.5
0.6
0.8
1.0
1.5
2.0
2.5
NC-1
PRTFDC1^OE-1
PRTFDC1^OE-2
PRTFDC1^OE-3
PRTFDC1^OE-4
